# Supplementary material for: Transient non-integrative expression of nuclear reprogramming factors promotes multifaceted amelioration of aging in human cells
Source: Nat Commun. 2020 Mar 24;11:1545. doi: 10.1038/s41467-020-15174-3 (PMC7093390; doi:10.1038/s41467-020-15174-3)
Supplement: Supplementary file 1 — Supplementary Information [file 41467_2020_15174_MOESM1_ESM.pdf]

Transient non-integrative expression of nuclear reprogramming factors promotes multifaceted amelioration of aging in human cells

Sarkar et al.

Supplementary Information

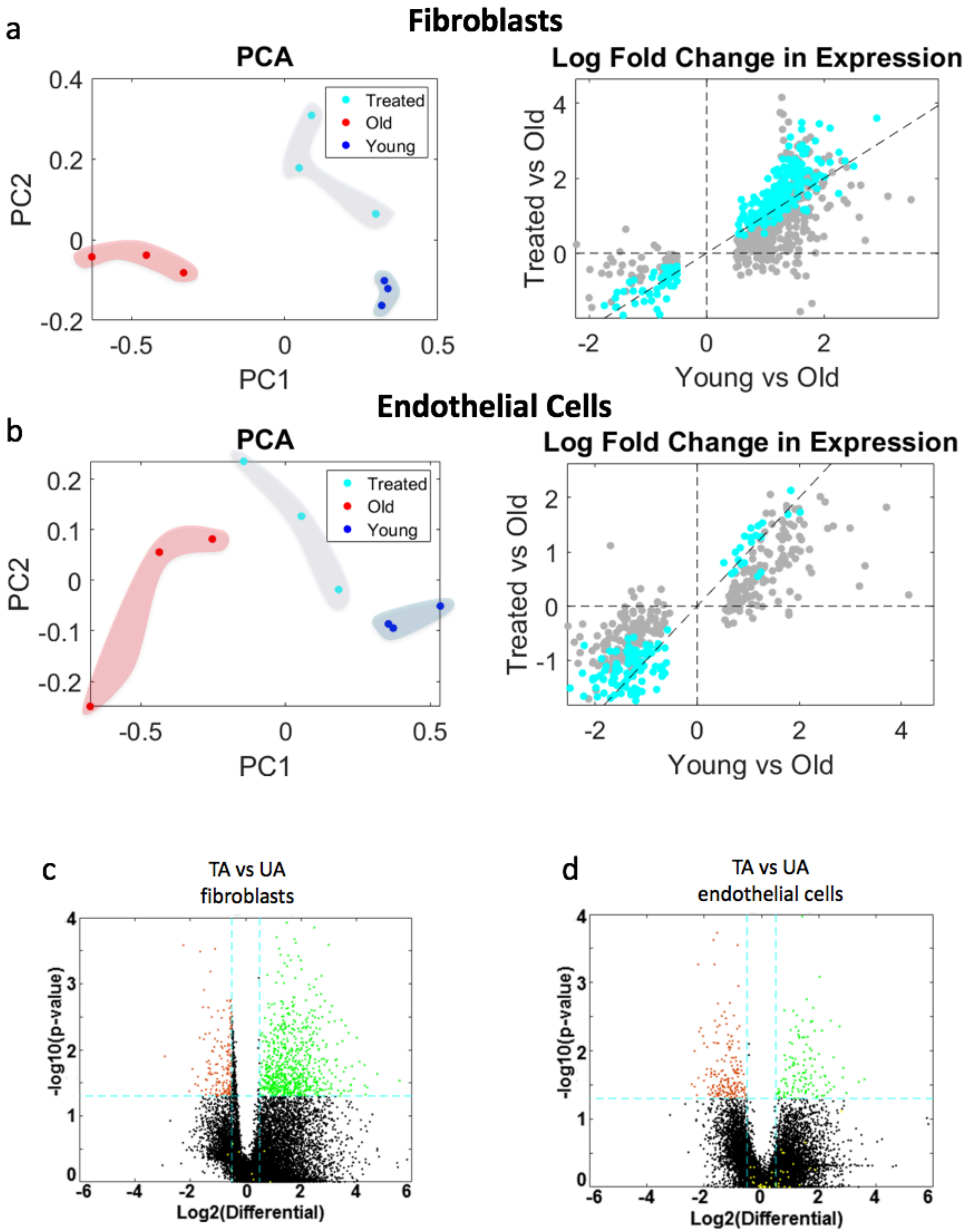

Figure S1: Supplement for Transcriptomic Analysis. a) Principal components analysis in the subspace defined by these differential genes showing clustering of treated and young fibroblasts away from the aged fibroblasts (Left) Comparison of log fold change between young and aged

(x-axis) and treated and aged (y-axis). Grey points are all the significant differential genes in the young vs aged comparison while cyan points are the genes that also meet significance criteria for treated vs aged comparison . A majority of the genes lie along y=x line, suggesting that the magnitude of changes by treatment closely matched the magnitude and the direction of change between young and aged. Significance is calculated with students t-test, pairwise between treated and aged, and groupwise when comparing to young patients. b) (Right) Principal components analysis in the subspace defined by these differential genes showing clustering of treated and young endothelial cells away from the aged endothelial cells. (Left) Comparison of log fold change between young and aged (x-axis) and treated and aged (y-axis). Grey points are all the significant differential genes in the young vs aged comparison while cyan points are the genes that also meet significance criteria for treated vs aged comparison . A majority of the genes lie along y=x line, suggesting that the magnitude of changes by treatment closely matched the magnitude and the direction of change between young and aged. Significance is calculated with students t-test, pairwise between treated and aged, and groupwise when comparing to young patients. c) Volcano plot showing treated vs aged fibroblast differential gene expression. d) Volcano plot showing treated vs aged endothelial cells differential gene expression.

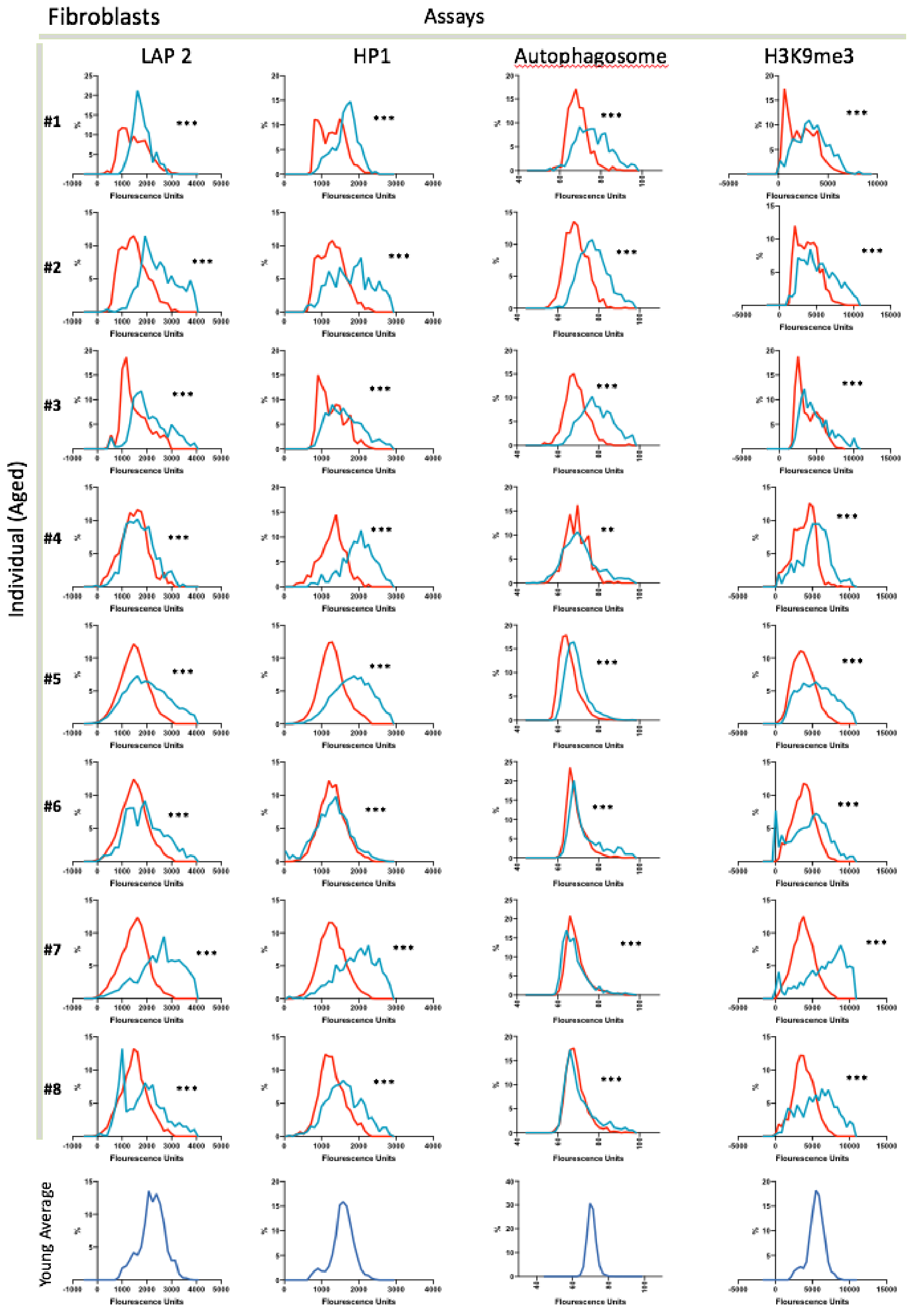

Figure S2: Single patient data distribution. Distribution of treated and untreated pairs of fibroblasts lines (treated: light blue, untreated: red), with pooled young controls (dark blue) for reference. Statistical analysis was conducted by t-test comparing treated and untreated cells. *= $p<0.05$; **= $p<0.01$; ***= $p<0.001$

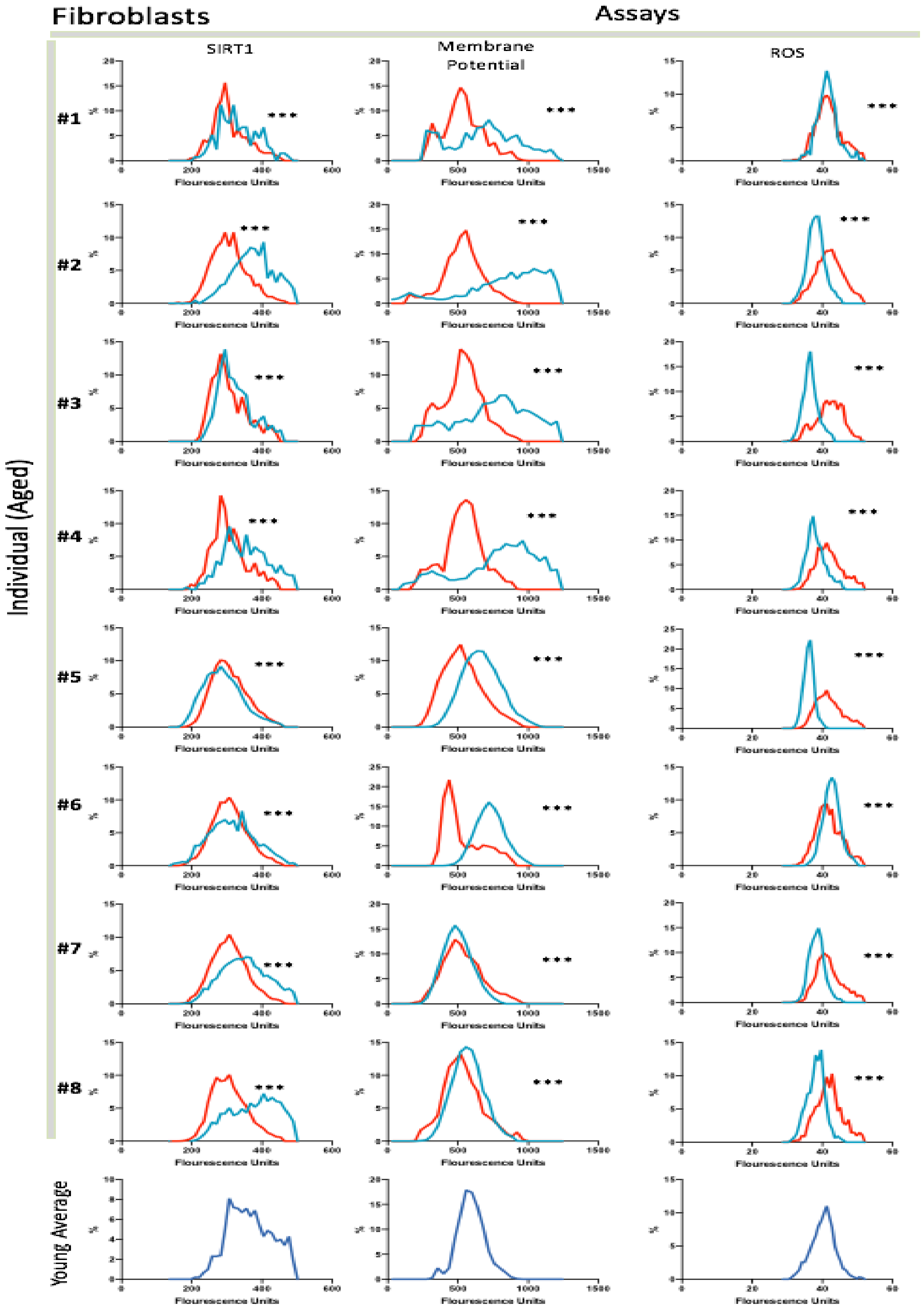

Figure S3: Single patient data distribution. Distribution of treated and untreated pairs of fibroblasts lines (treated: light blue, untreated: red), with pooled young controls (dark blue) for reference. Statistical analysis was conducted by t-test comparing treated and untreated cells. *= p<0.05; **=p<0.01;***=p<0.001

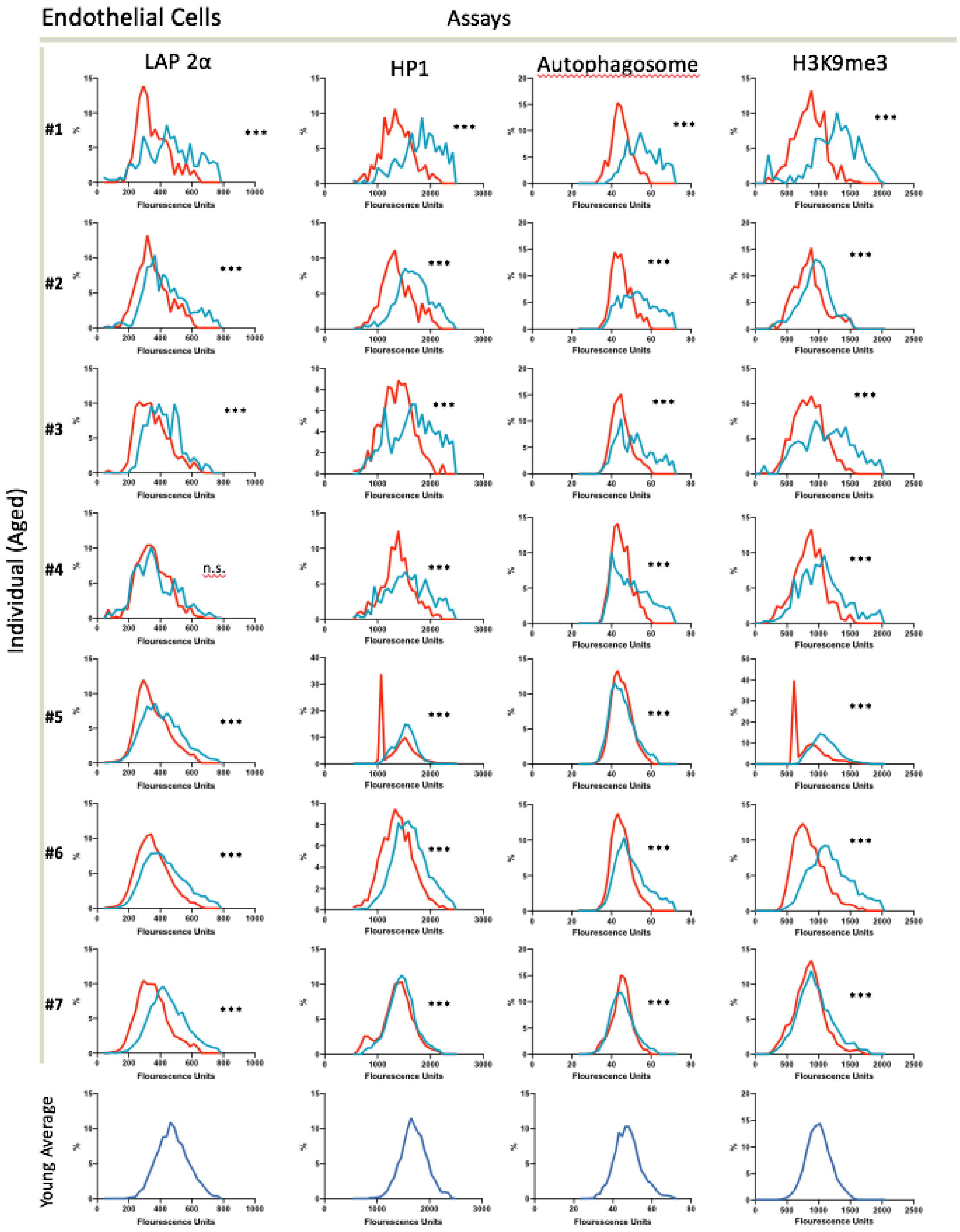

Figure S4: Single patient data distribution. Distribution of treated and untreated pairs of endothelial cells lines (treated: light blue, untreated: red), with pooled young controls (dark blue) for reference. Statistical analysis was conducted by t-test comparing treated and untreated cells. *= $p<0.05$; **=$p<0.01$;***=$p<0.001$

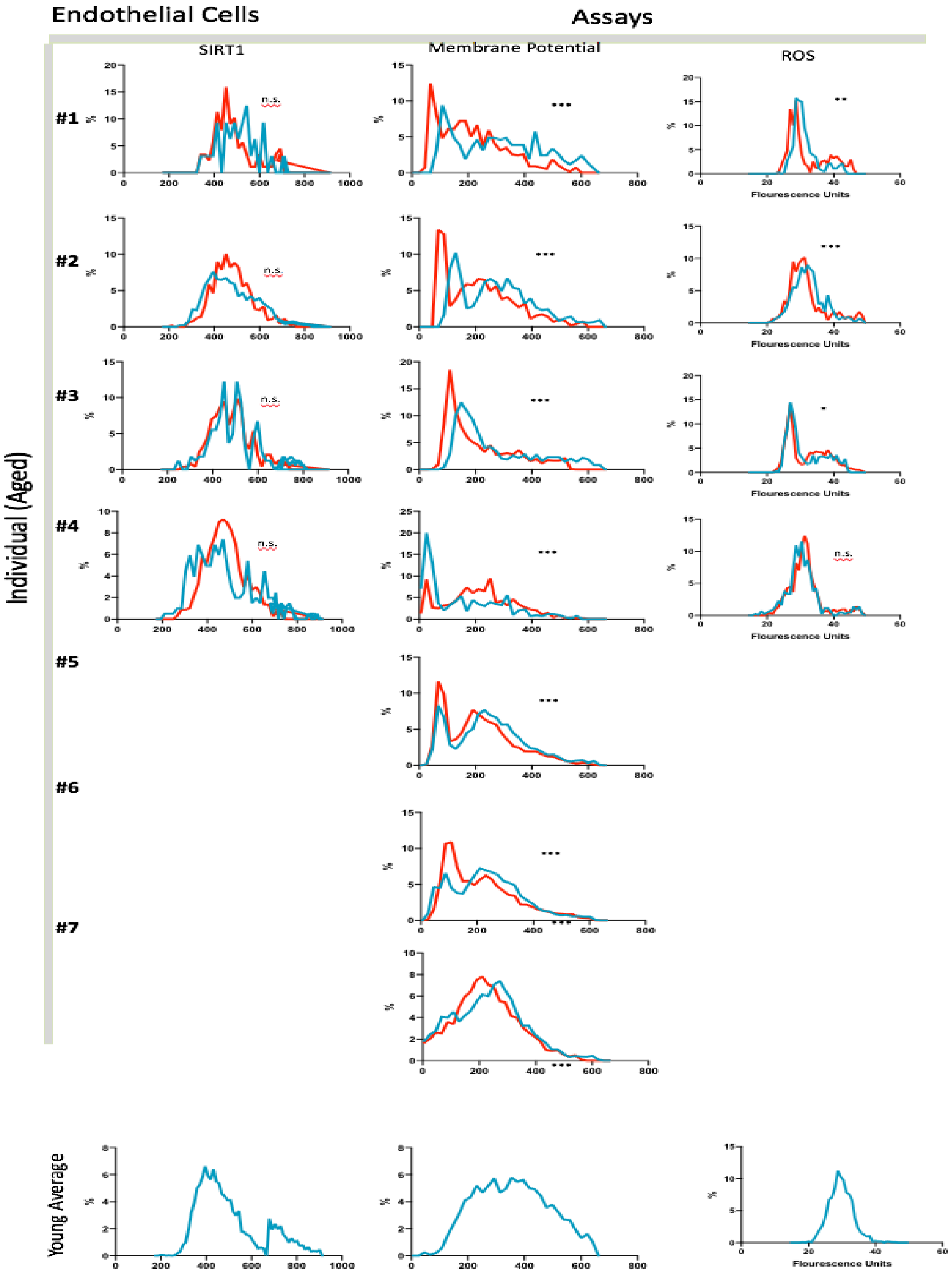

Figure S5: Single patient data distribution. Distribution of treated and untreated pairs of endothelial cells lines (treated: light blue, untreated: red), with pooled young controls (dark blue) for reference. Statistical analysis was conducted by t-test comparing treated and untreated cells.
*= p<0.05; **=p<0.01;***=p<0.001

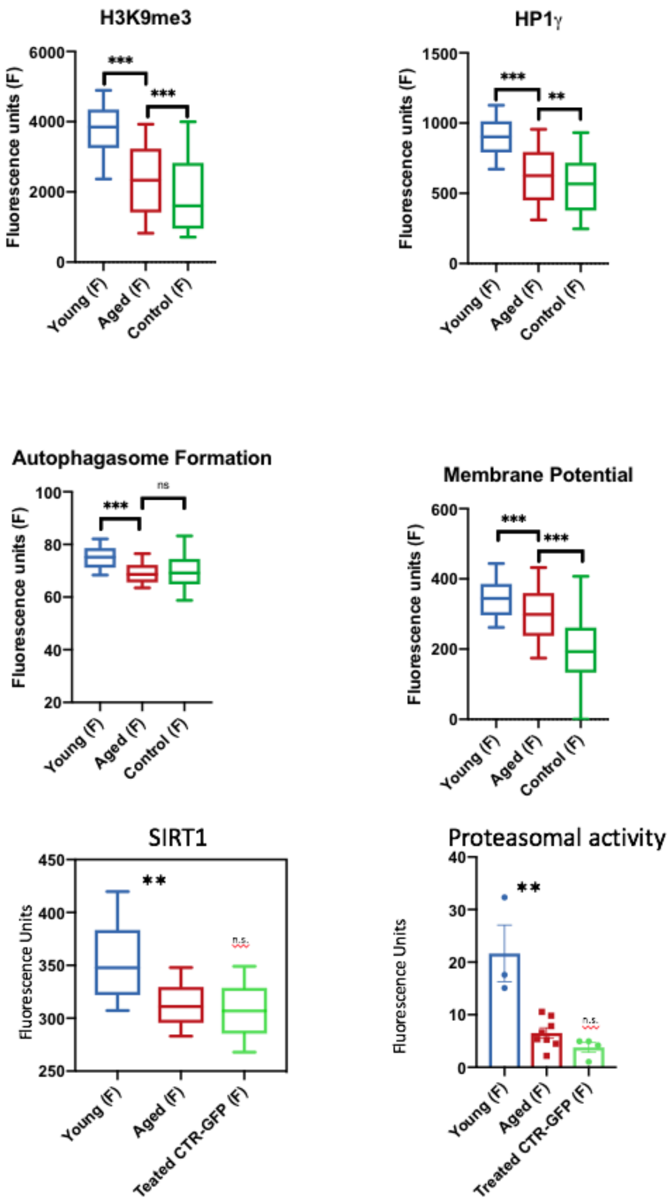

Figure S6: GFP transfection control in Fibroblasts: Comparison between untreated young and aged fibroblasts vs fibroblasts transfected with GFP mRNA; results show that GFP mRNA results in no alteration or worsening of the hallmarks of aging. Analysis for H3K9me3, HP1-gamma, LAP2alpha, Autophagosome, Membrane potential, Mitochondrial ROS and Sirtuin was conducted by high throughput imaging on 500-1000 cells per sample (as shown in Supplementary Figures 2-5) to allow population-wide studies with single cell resolution. 100 cells per sample were randomly selected to do a statistical comparison across the three groups. Statistical analysis was then done by ANOVA, followed by pairwise comparisons done with t-tests with correction for multiple comparisons. P values: *<.05, **<.01, ***<.001. Young n=3; aged n=8; aged treated n=8. In Proteosomal activity error bars represent s.d. In all the other panels distributions are represented as box and whisker plots with whisker depicting 10th and 90th percentile.

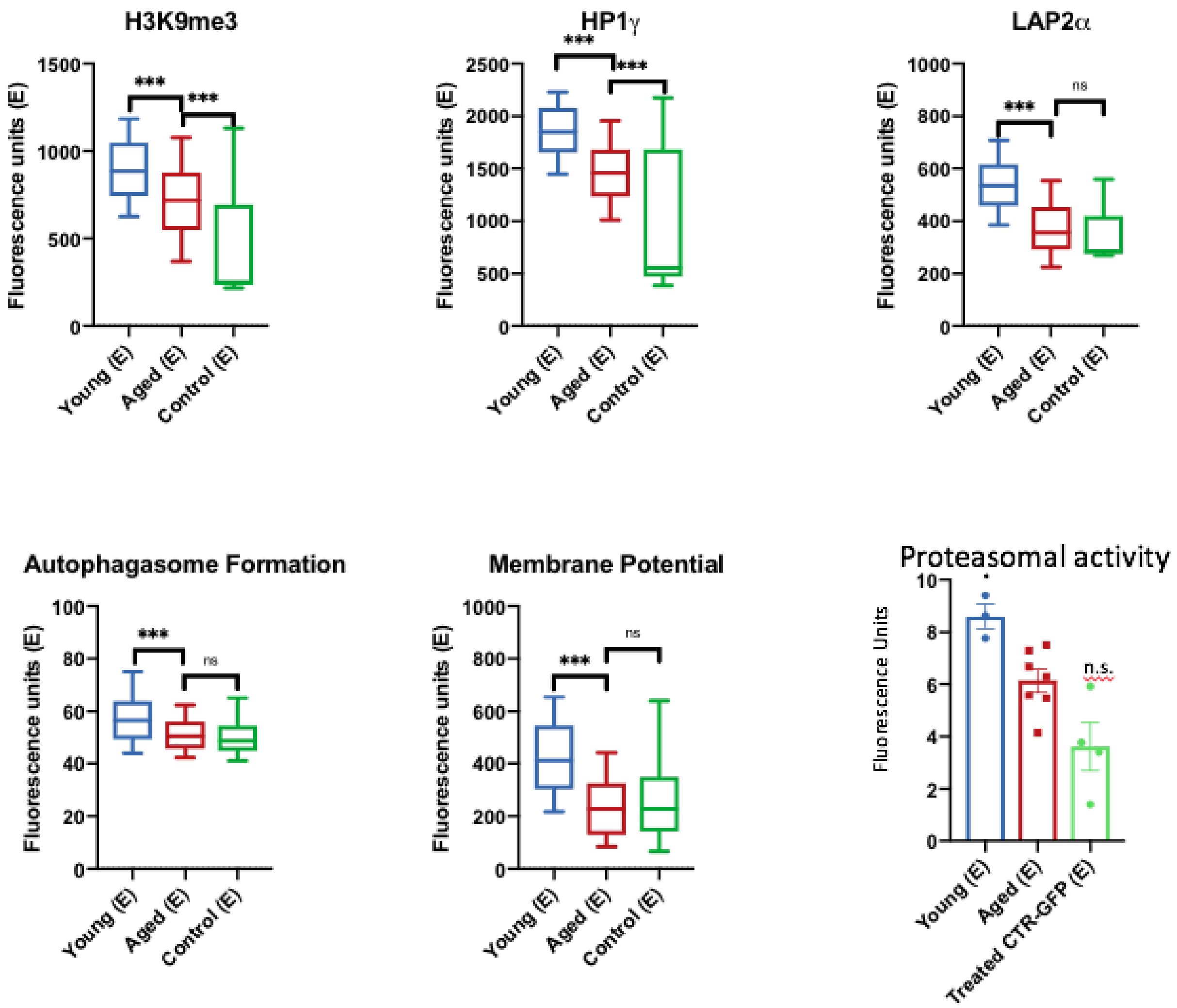

Figure S7: GFP transfection control in Endothelial Cells. Comparison between untreated young and aged endothelial cells vs endothelial cells transfected with GFP mRNA; results show that GFP mRNA results in no alteration or worsening of the hallmarks of aging.

Analysis for H3K9me3, HP1-gamma, LAP2alpha, Autophagosome, Membrane potential, was conducted by high throughput imaging on 500-1000 cells per sample to allow population-wide studies with single cell resolution. 100 cells per sample were randomly selected to do a statistical comparison across the three groups. Statistical analysis was then done by ANOVA, followed by pairwise comparisons done with t-tests with correction for multiple comparisons. P values: *<.05, **<.01, ***<.001.lP values: *<.05, **<.01, ***<.001. Statistical anlysis by one-way ANOVA was conducted for all the other assays.

Young n=3; aged n=7; aged treated n=7.

In Proteosomal activity error bars represent s.d. In all the other panels distributions are represented as box and whisker plots with whisker depicting 10th and 90th percentile.

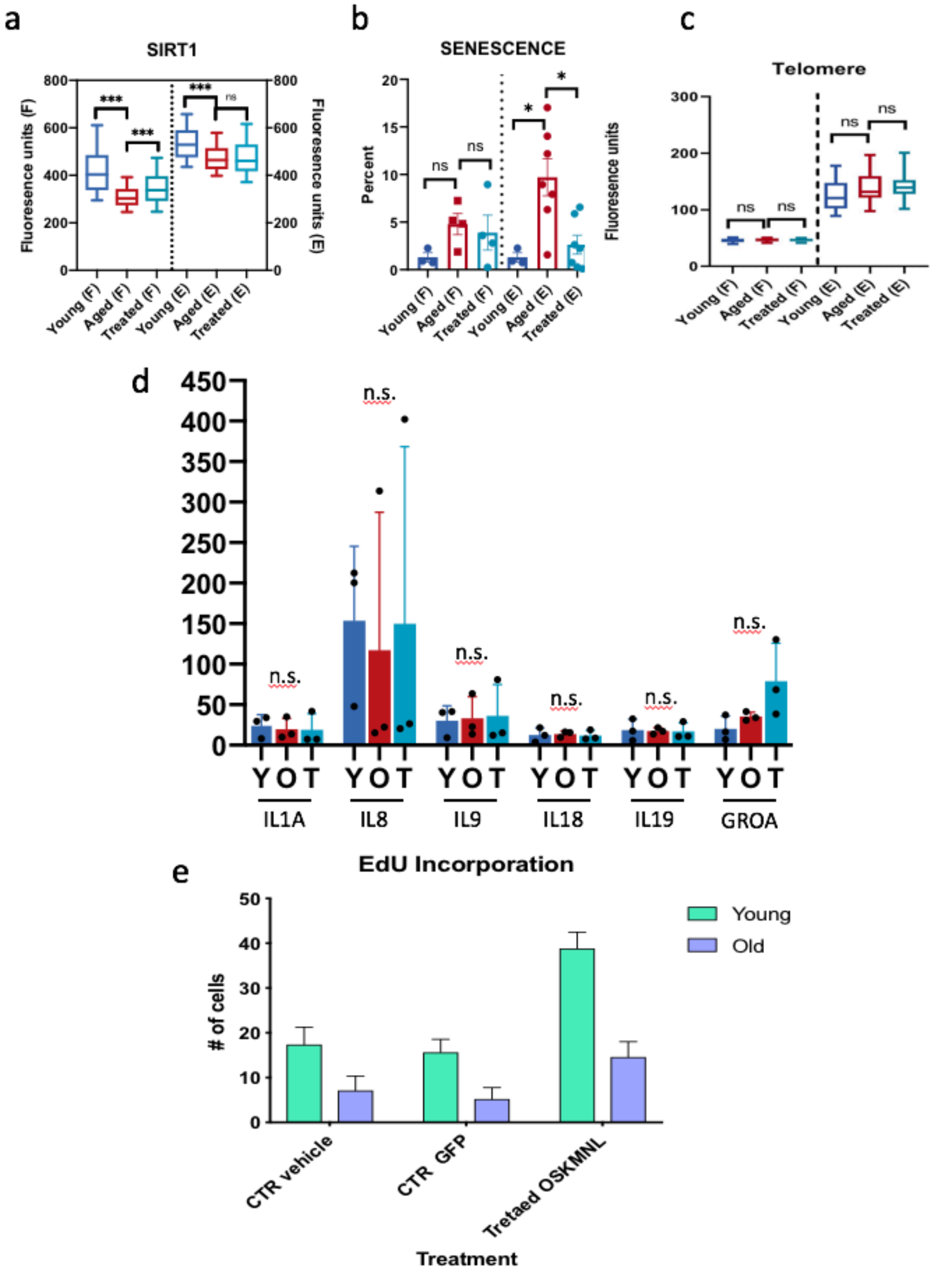

Figure S8. Transient reprogramming reverts aged physiology towards a more youthful state in human fibroblasts and endothelial cells: a) SIRT1 expression significantly increases with treatment in fibroblasts. b) diminishment in senescent percentage in endothelial cell population with treatment. c) no significant change in telomere length in either cell type with treatment. D) Cytokines profile shows no difference between young, aged and aged treated fibroblasts, likely due to the fact that no difference was observed in numbers of senescent cells. e) EdU incorporation assay in young and old fibroblasts showing increased cellular proliferation upon treatment with OSKMNL). Analysis for panels a and c was conducted by high throughput imaging on 500-1000 cells per sample to allow population-wide studies with single cell resolution. 100 cells per sample were randomly selected to do a statistical comparison across the three groups. Statistical analysis was then done by ANOVA, followed by pairwise comparisons

done with t-tests with correction for multiple comparisons. P values: *<.05, **<.01, ***<.001.lP values: *<.05, **<.01, ***<.001. Statistical anlysis by one-way ANOVA was conducted for all the other assays. Young n=3; aged n=8; aged treated n=8. In Senescence and EdU incorporation plot activity error bars represent s.d. In all the other panels distributions are represented as box and whisker plots with whisker depicting 10th and 90thpercentile.

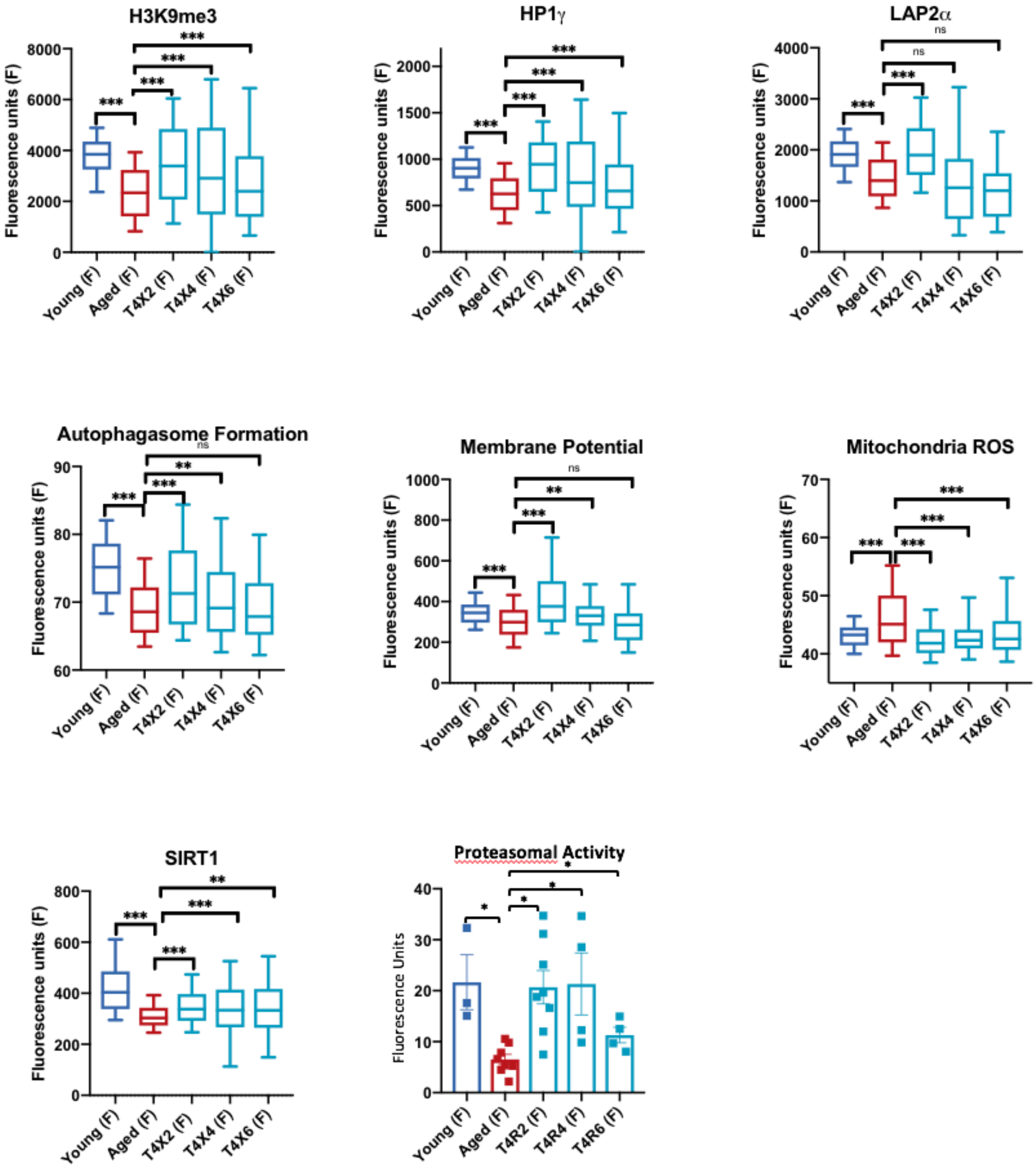

Figure S9. Perdurance of Effect in Fibroblasts: Extending out the observation period after treatment shows a retention of most effects on into 4 and 6 days after treatment is ceased in fibroblasts (T#R$ denotes Treated # days and Observed $ days later). Analysis was conducted by high throughput imaging on 500-1000 cells per sample (as in Supplementary Figures 2-5) to allow population-wide studies with single cell resolution. 100 cells per sample were randomly selected to do a statistical comparison across the groups. Statistical analysis was then done by ANOVA, followed by pairwise comparisons done with t-tests with correction for multiple comparisons. P values: *<.05, **<.01, ***<.001.lP values: *<.05, **<.01, ***<.001. Statistical anlysis by one-way ANOVA was conducted for all the other assays. Young n=3; aged n=8; aged treated n=8. In Proteosomal activity error bars represent s.d. In all the other panels distributions are represented as box and whisker plots with whisker depicting 10th and 90th percentile.

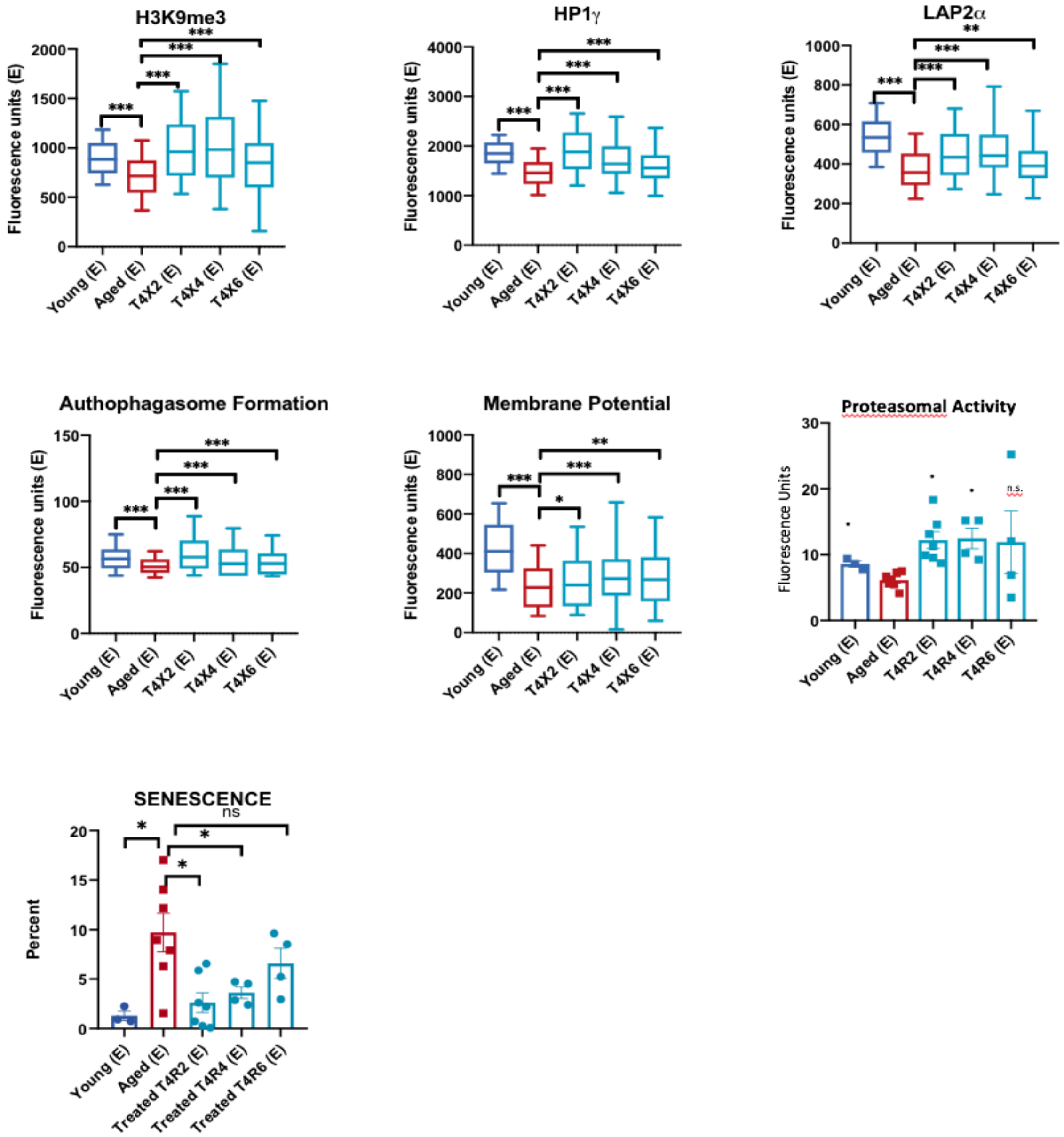

Figure S10. Perdurance of Effect Endothelial Cells: Extending out the observation period after treatment shows a retention of most effects on into 4 and 6 days after treatment is ceased in endothelial cells (T#R$ denotes Treated # days and Observed $ days later). Analysis was conducted by high throughput imaging on 500-1000 cells per sample (as in Supplementary Figures 2-5) to allow population-wide studies with single cell resolution. 100 cells per sample were randomly selected to do a statistical comparison across the groups. Statistical analysis was then done by ANOVA, followed by pairwise comparisons done with t-tests with correction for multiple comparisons. P values: *<.05, **<.01, ***<.001.lP values: *<.05, **<.01, ***<.001. Statistical anlysis by one-way ANOVA was conducted for all the other assays. Young n=3; aged n=7; aged treated n=7. In Proteosomal activity and senescence error bars represent s.d. In all the other panels distributions are represented as box and whisker plots with whisker depicting 10th and 90th percentile.

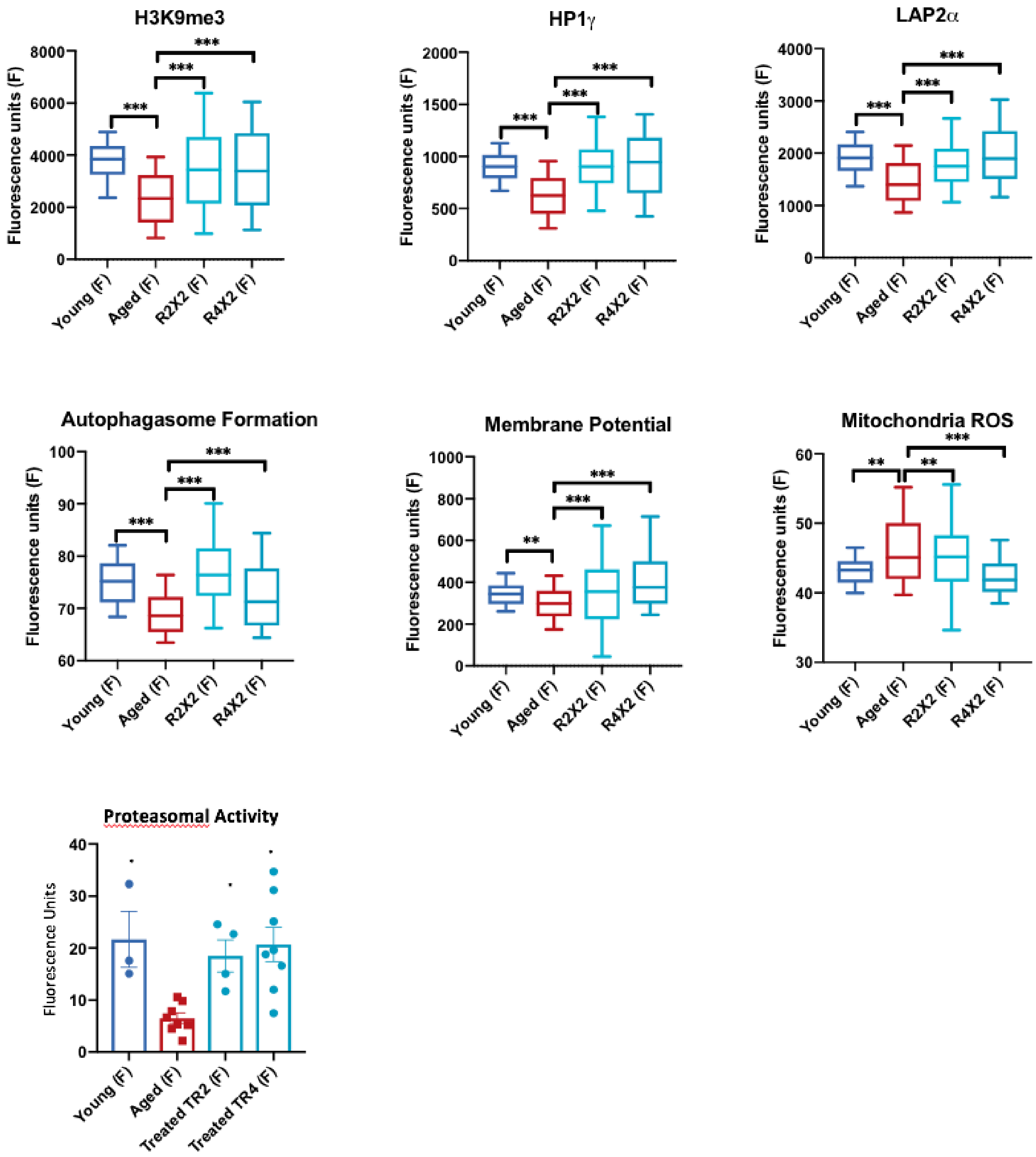

Figure S11. Treatment Options in Fibroblasts: Comparing different duration of treatment shows early effects with 2 days treatment that are more substantial with 4 days treatment in fibroblasts (T#R$ denotes Treated # days and Observed $ days later). Analysis was conducted by high throughput imaging on 500-1000 cells per sample (as in Supplementary Figures 2-5) to allow population-wide studies with single cell resolution. 100 cells per sample were randomly selected to do a statistical comparison across the groups. Statistical analysis was then done by ANOVA, followed by pairwise comparisons done with t-tests with correction for multiple comparisons. P values: *<.05, **<.01, ***<.00.1Young n=3; aged n=8; aged treated n=8.
In Proteosomal activity error bars represent s.d. In all the other panels distributions are represented as box and whisker plots with whisker depicting 10th and 90th percentile.

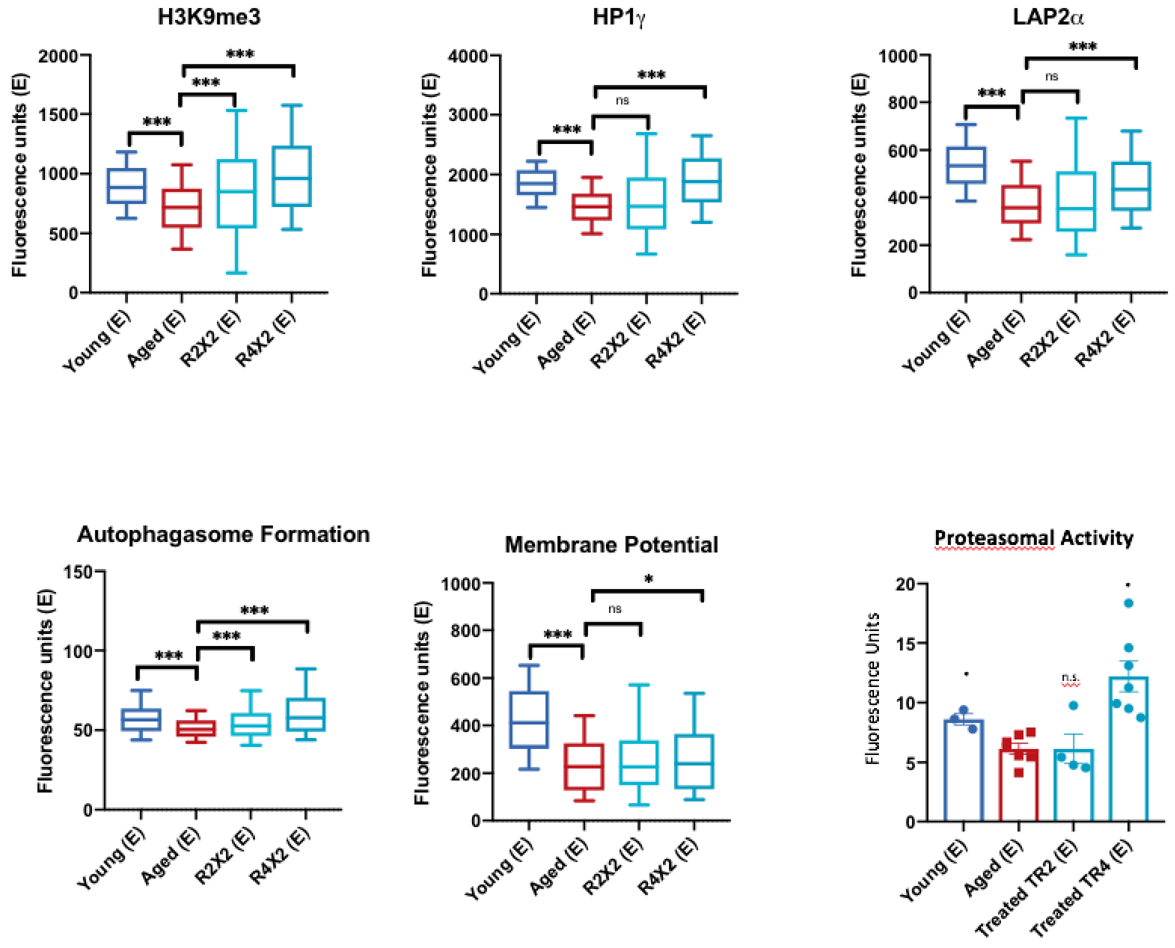

Figure S12. Treatment Options Endothelial Cells: Comparing different duration of treatment shows early effects with 2 days treatment that are more substantial with 4 days treatment in endothelial cells (T#R$ denotes Treated # days and Observed $ days later). Analysis was conducted by high throughput imaging on 500-1000 cells per sample (as in Supplementary Figures 2-5) to allow population-wide studies with single cell resolution. 100 cells per sample were randomly selected to do a statistical comparison across the three groups. Statistical analysis was then done by ANOVA, followed by pairwise comparisons done with t-tests with correction for multiple comparisons. P values: *<.05, **<.01, ***<.001.lP values: *<.05, **<.01, ***<.001. Statistical anlysis by one-way ANOVA was conducted for all the other assays. Young n=3; aged n=7; aged treated n=7. In Proteosomal activity error bars represent s.d. In all the other panels distributions are represented as box and whisker plots with whisker depicting 10th and 90th percentile.

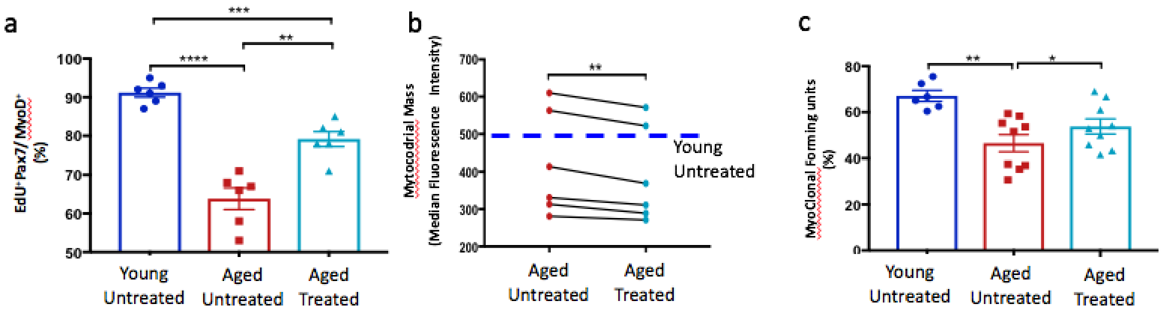

Figure S13. Transient reprogramming promotes youthful phenotypes in aged MuSCs. a) Partial reprogramming restores slower kinetics of activation from quiescence of aged MuSCs. EDU staining reveals greater percentage of young and aged treated activated MuSCs compared to aged untreated MuSCs after switching from 24 hours from quiescence media to growth media (n=6). b) Partial reprogramming restores mitochondrial mass increased with age in MuSCs. Mitotracker staining reveals less mitochondrial mass in aged treated MuSCs and young MuSCs compared to aged untreated MuSCs (n=6). c) Partial reprogramming restores unpaired myoclonal formation *in vitro* from aged MuSCs. Percent of myogenic colony forming cells is higher in young untreated and aged treated single MuSCs (Young Untreated, n=6; Aged Untreated and Aged Treated, n=9). Error bars represent SD.

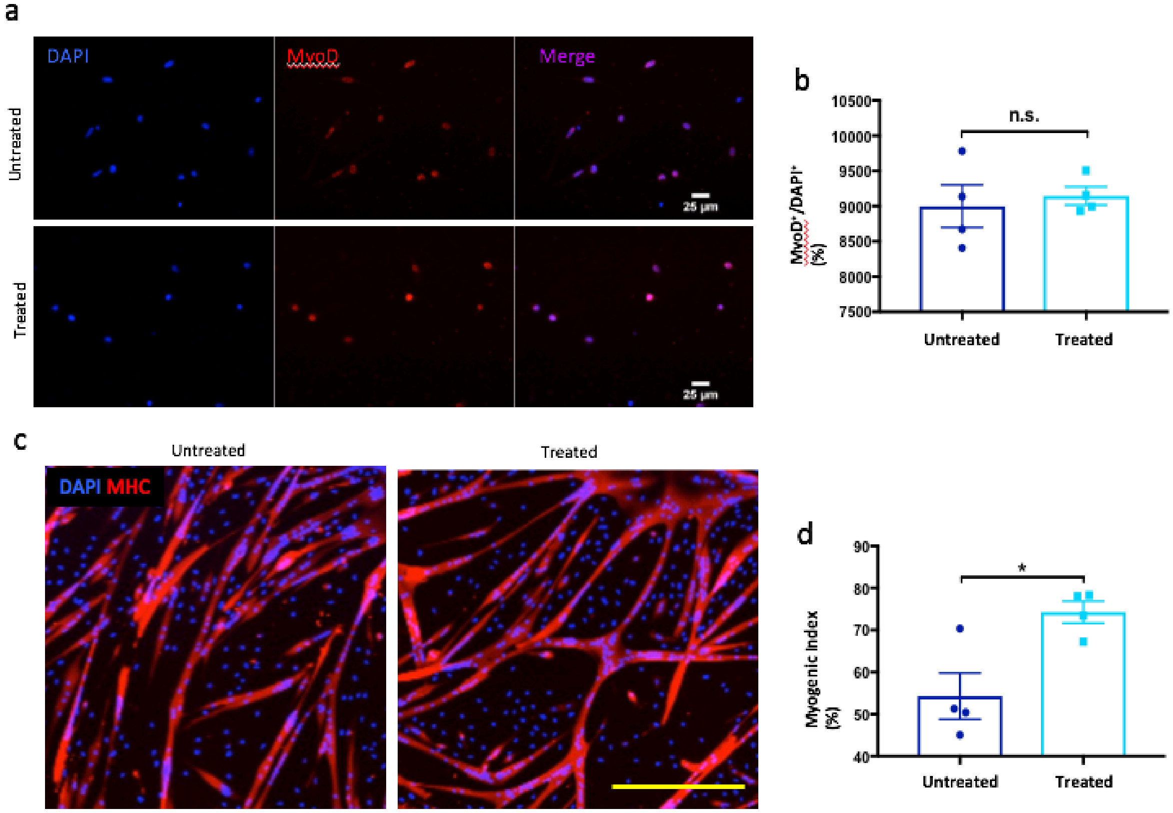

Figure S14. Transient reprogramming does not alter myogenic fate and improves the myogenic potential of MuSCs in vitro a) Representative immunofluorescence images of cultured transient reprogrammed mouse MuSCs. Cells were immunostained with antibodies against MyoD (red). b) Quantification of immunofluorescence staining in (a). c) Representative immunofluorescence images of cultured transient reprogrammed mouse MuSCs. Cells were immunostained with antibodies against Myosin Heavy Chain (MHC) (red). d) Quantification of immunofluorescence staining in (c). Aged untreated n=4; aged treated n=4. Error bars represent SD.

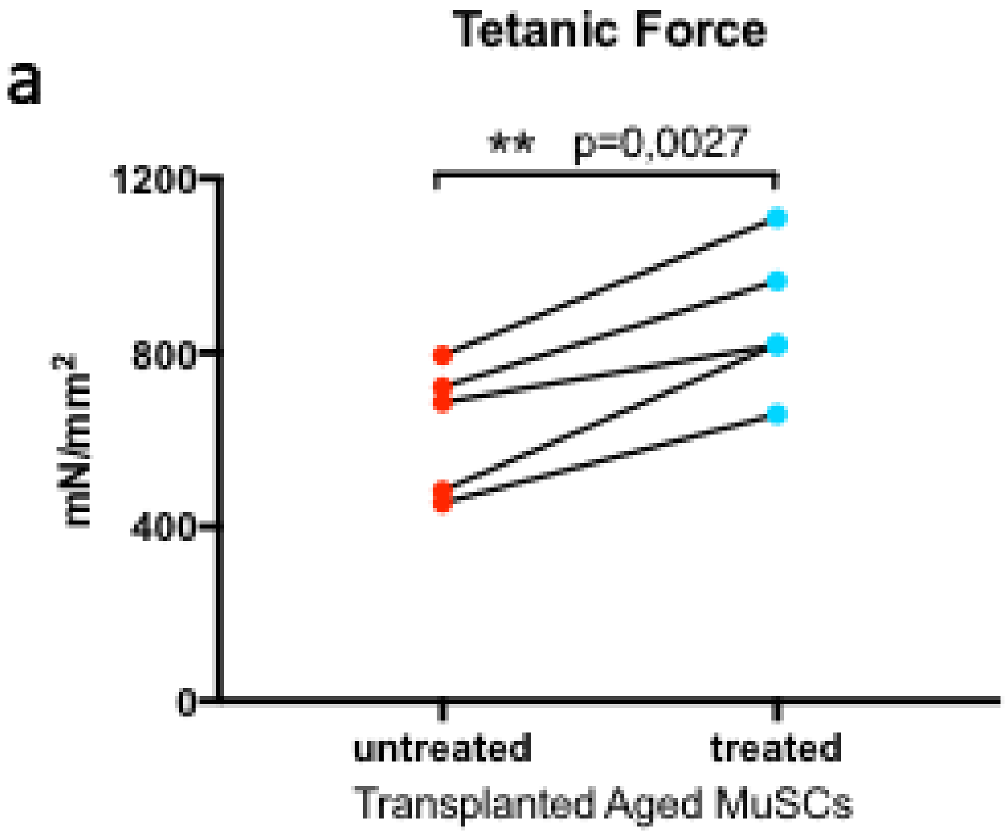

Figure S15. Transient reprogrammed transplanted aged MuSCs improves tetanic forces in aged mice. a) Tetanic force measurements of aged muscles transplanted with aged MuSCs. TA muscles were dissected and electrophysiology ex vivo for tetanic measurement performed. Treated aged MuSCs were transplanted into TA muscles of aged mice and force production measured 30 days later. The graphic shows paired analysis of contralateral TA muscles transplanted with aged MuSCs derived from the same donor and either untreated or treated with transient reprogramming (n=5).

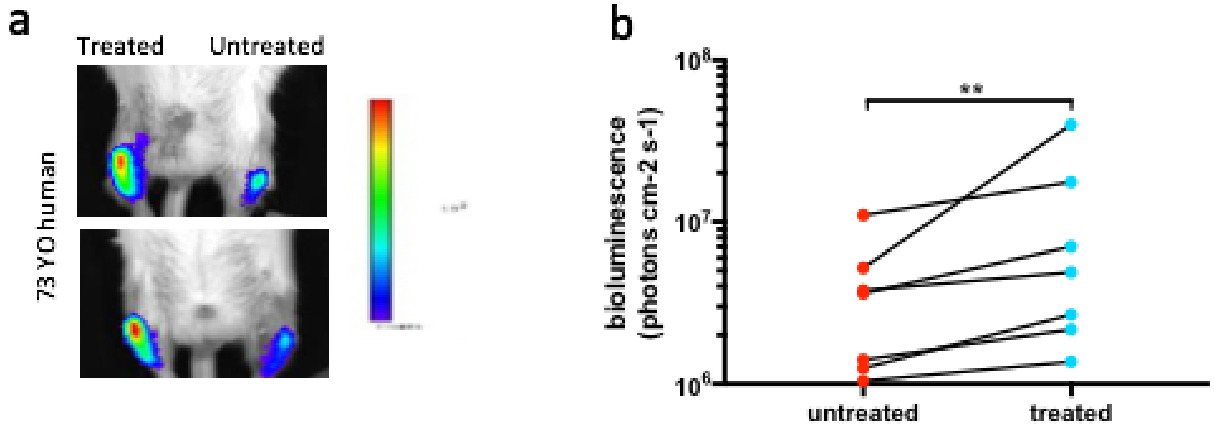

Figure S16. Transient reprogramming enhances transplantation potential of human MuSCs a) Representative images of bioluminescence measured from mice 11 days after transplantation in TA muscles of treated/untreated Luciferase^{+} human aged MuSCs. b) Quantified results of bioluminescence in (a) 7 days following injury and transplantation of aged human MuSCs (donor age group: 60-80 years old) (n=7).

Transplanted Cell Type	Teratomas	Solid Tumors	Hematopoietic Tumors
Untreated Young MuSCs	0	0	0
Untreated Aged MuSCs	0	0	0
Treated Aged MuSCs	0	0	0

Supplementary Table 1. Neoplastic assessment 3 months following cell transplantation in mice. Autoptic samples were dissected and analyzed under the microscope for anatomical and histological evidence of tumor formation (n=10).
