# Supplementary material for: Transient non-integrative expression of nuclear reprogramming factors promotes multifaceted amelioration of aging in human cells
Source: Nat Commun. 2020 Mar 24;11:1545. doi: 10.1038/s41467-020-15174-3 (PMC7093390; doi:10.1038/s41467-020-15174-3)
Supplement: Supplementary file 3 — Reporting Summary [file 41467_2020_15174_MOESM3_ESM.pdf]

nature research

Corresponding author(s): Vittorio Sebastiano

Last updated by author(s): *YYYY-MM-DD*

Reporting Summary

Nature Research wishes to improve the reproducibility of the work that we publish. This form provides structure for consistency and transparency in reporting. For further information on Nature Research policies, see Authors & Referees and the Editorial Policy Checklist.

Statistics

For all statistical analyses, confirm that the following items are present in the figure legend, table legend, main text, or Methods section.

n/a	Confirmed	
☐	☒	The exact sample size (n) for each experimental group/condition, given as a discrete number and unit of measurement
☐	☒	A statement on whether measurements were taken from distinct samples or whether the same sample was measured repeatedly
☐	☒	The statistical test(s) used AND whether they are one- or two-sided<br>*Only common tests should be described solely by name; describe more complex techniques in the Methods section.*
☐	☒	A description of all covariates tested
☐	☒	A description of any assumptions or corrections, such as tests of normality and adjustment for multiple comparisons
☐	☒	A full description of the statistical parameters including central tendency (e.g. means) or other basic estimates (e.g. regression coefficient) AND variation (e.g. standard deviation) or associated estimates of uncertainty (e.g. confidence intervals)
☐	☒	For null hypothesis testing, the test statistic (e.g. F, t, r) with confidence intervals, effect sizes, degrees of freedom and P value noted<br>*Give P values as exact values whenever suitable.*
☒	☐	For Bayesian analysis, information on the choice of priors and Markov chain Monte Carlo settings
☒	☐	For hierarchical and complex designs, identification of the appropriate level for tests and full reporting of outcomes
☒	☐	Estimates of effect sizes (e.g. Cohen's d, Pearson's r), indicating how they were calculated

Our web collection on statistics for biologists contains articles on many of the points above.

Software and code

Policy information about availability of computer code

Data collection	N/A
Data analysis	N/A

For manuscripts utilizing custom algorithms or software that are central to the research but not yet described in published literature, software must be made available to editors/reviewers. We strongly encourage code deposition in a community repository (e.g. GitHub). See the Nature Research guidelines for submitting code & software for further information.

Data

Policy information about availability of data

All manuscripts must include a data availability statement. This statement should provide the following information, where applicable:

- Accession codes, unique identifiers, or web links for publicly available datasets
- A list of figures that have associated raw data
- A description of any restrictions on data availability

The data that support the findings of this study are available from the corresponding author upon request.

Field-specific reporting

Please select the one below that is the best fit for your research. If you are not sure, read the appropriate sections before making your selection.

☒ Life sciences ☐ Behavioural & social sciences ☐ Ecological, evolutionary & environmental sciences

For a reference copy of the document with all sections, see nature.com/documents/nr-reporting-summary-flat.pdf

Life sciences study design

All studies must disclose on these points even when the disclosure is negative.

Sample size	no sample size calculation was applied. The number was determined to be as high as possible in particular for the aged population (>6)
Data exclusions	no data exclusion was applied
Replication	The experiment were reproduced by multiple experimenter independently to confirm the reproducibility of the findings.
Randomization	N/A
Blinding	N/A

Reporting for specific materials, systems and methods

We require information from authors about some types of materials, experimental systems and methods used in many studies. Here, indicate whether each material, system or method listed is relevant to your study. If you are not sure if a list item applies to your research, read the appropriate section before selecting a response.

Materials & experimental systems

n/a	Involved in the study
☐	☒ Antibodies
☐	☒ Eukaryotic cell lines
☒	☐ Palaeontology
☐	☒ Animals and other organisms
☒	☐ Human research participants
☒	☐ Clinical data

Methods

n/a	Involved in the study
☒	☐ ChIP-seq
☒	☐ Flow cytometry
☒	☐ MRI-based neuroimaging

Antibodies

Antibodies used	The following antibodies were used in this study. The source of each antibody is indicated. Rabbit:: H3K9me3 (Abcam #ab8898 1: 4000) , LAP2α (Abcam #ab5162 1:500), SIRT1 (Abcam #ab7343 1:200); Rabbit: Mouse: HP1γ (Millipore Sigma #05-690 1:200), Lamin A/C (Abcam #ab40567 1:200), GFP (Invitrogen, #A11122, 1:250); Luciferase (Sigma-Aldrich, #L0159, 1:200); Collagen I (Cedarlane Labs, #CL50151AP, 1:200); HSP47 (Abcam, #ab77609, 1:200), Laminin (Abcam, #AB11576, 1:1000), anti-CD31-Alexa Fluor 488 (clone WM59; BioLegend; #303110, 1:75), anti-CD45-Alexa Fluor 488 (clone HI30; Invitrogen; #MHCD4520, 1:75), anti-CD34-FITC (clone 581; BioLegend; #343503, 1:75), anti-CD29-APC (clone TS2/16; BioLegend; #303008, 1:75) and anti-NCAM-Biotin (clone HCD56; BioLegend; #318319, 1:75), anti- CD31-Alexa Fluor 488 (clone WM59; BioLegend; #303110, 1:75), anti-CD45-Alexa Fluor 488 (clone HI30; Invitrogen; #MHCD4520, 1:75), anti-CD34-FITC (clone 581; BioLegend; #343503, 1:75), anti-CD29-APC (clone TS2/16; BioLegend; #303008, 1:75) and anti-NCAM-iotin (clone HCD56; BioLegend; #318319, 1:75).
Validation	Antibodies were used according to the manufacturer's instructions and guidelines

Eukaryotic cell lines

Policy information about cell lines

Cell line source(s)	Endothelial cells and four of the fibroblasts lines were obtained from Coriell Repository. Theremaining four fibrobalsts lines were obtained from the laboratory of Tony Wyss Coray.
Authentication	None of the cell lines were authenticated
Mycoplasma contamination	All the cell lines used in the study were tested and confirmed to be Mycoplasma Free
Commonly misidentified lines (See ICLAC register)	N/A

Animals and other organisms

Policy information about studies involving animals; ARRIVE guidelines recommended for reporting animal research

Laboratory animals	C57BL/6 male mice and NSG mice were obtained from Jackson Laboratory. NOD/MrkBomTac-Prkdcscid mice were obtained from Taconic Biosciences. Mice were housed and maintained in the Veterinary Medical Unit at the Veterans Affairs Palo Alto Health Care Systems.

Wild animals	N/A
Field-collected samples	N/A
Ethics oversight	The Administrative Panel on Laboratory Animal Care of Stanford University approved animal protocols.

Note that full information on the approval of the study protocol must also be provided in the manuscript.
