# Supplementary material for: Transient non-integrative expression of nuclear reprogramming factors promotes multifaceted amelioration of aging in human cells
Source: Nat Commun. 2020 Mar 24;11:1545. doi: 10.1038/s41467-020-15174-3 (PMC7093390; doi:10.1038/s41467-020-15174-3)
Supplement: Supplementary file 4 — Description of Additional Supplementary Files [file 41467_2020_15174_MOESM4_ESM.pdf]

Description of Additional Supplementary Files

File Name: Supplementary Data 1
Description: Y vs A gene signature in Fibroblasts

File Name: Supplementary Data 2
Description: Y vs A gene signature in Endothelial Cells

File Name: Supplementary Data 3
Description: MSigDB Y vs A Fibroblasts

File Name: Supplementary Data 4
Description: MSigDB Y vs A Endothelial Cells

File Name: Supplementary Data 5
Description: T vs A gene signature in Fibroblasts

File Name: Supplementary Data 6
Description: T vs A gene signature in Endothelial Cells

File Name: Supplementary Data 7
Description: MSigDB T vs A Fibroblasts

File Name: Supplementary Data 8
Description: MSigDB T vs A Endothelial Cells

File Name: Supplementary Data 9
Description: Overlap Signature in Fibroblasts

File Name: Supplementary Data 10
Description: Overlap Signature in Endothelial Cells

File Name: Supplementary Data 11
Description: Cell type specific markers

File Name: Supplementary Data 12
Description: Hallmarks of aging
